# Clinical trial data reuse – overcoming complexities in trial design and data sharing

**DOI:** 10.1186/s13063-019-3627-6

**Published:** 2019-08-19

**Authors:** Toby Wilkinson, Siddharth Sinha, Niels Peek, Nophar Geifman

**Affiliations:** Centre for Health Informatics, Division of Informatics, Imaging, and Data Sciences, Faculty of Biology, Medicine and Health, University of Manchester, Manchester, UK

**Keywords:** Data sharing, Clinical trials, Pooled analysis

## Abstract

There are many acknowledged benefits for the reuse of clinical trial data; from independent verification of published results to the evaluation of new hypotheses. However, the reuse of shared clinical trial data is not without obstacles. Here we present some of the issues and lessons learned from our own experiences in accessing and analyzing trial data; specifically, where we aim to combine and pool data from multiple different trials. In addition to issues around missing annotation and incomplete datasets, we identify trial-design complexity as a potential hurdle that may complicate downstream analyses. We address potential solutions and emphasize the need for benefits of transparent sharing and analysis of participant-level clinical trial data with appropriate risk mitigation, a matter important to efficient clinical research.

## Background: Open sharing of clinical trial data

Data sharing is well recognized as crucial for transparent, reproducible research; yet, best practices for the means, timing, and governance under which trial data are shared are still under question [1–3]. A recent evaluation of issues related to the sharing of patient-level clinical trial data produced practical recommendations for such sharing from non-commercial trials [4]. Other examples for good practice exist; specifically, with regards to the sharing of non-trial research data [5]. In other areas, such as with electronic health records data, mechanisms and principles for standardization have been developed and widely accepted. For example, the Fast Health Interoperability Resources (FHIR) stipulates that data should be Findable, Accessible, Interoperable, and Reusable, and emphasizes the need for machine-automated capabilities, in addition to supporting data reuse by individuals.

While the benefits of open sharing of clinical trial data are recognized by many in the medical research and health-provider communities, the debate on benefits and risks for granting free access to such data is ongoing [1, 6, 7] with different modes of access proposed as possible solutions to some of the related concerns [8, 9]. The various stakeholder groups within the clinical trials community each face their own set of risks. One such potential risk is that secondary analysts, i.e. researchers not originally involved in the study, will not have full understanding of the trial design, the cohort of recruited patients, and, hence, the underlying complexities in the data [10]. Erroneous interpretations of the data, which may challenge the original findings, then becomes a real risk to both the primary executors of the trial as well as the downstream patients. Here we describe a few of the challenges in the combining and pooling of shared clinical trial data for secondary analysis, from the perspective of a data reuser. Specifically, we focus on issues related to the quality of the data being shared, and to differences in the design of the trials being reused in a pooled analysis.

## Transparency and availability of information

Lack of clarity around the availability of data often poses a barrier for secondary use of trial data. Often, trial repositories provide a short description of the trial setting, objectives, and design; data dictionaries and schemas that describe the exact content of the datasets are not always provided in advance of access to the data, and on occasion are incomplete. Further, in some instances, data that should be available, are not included in the release.

In our attempt to assess and compare the outcomes of three standards of care in breast cancer, careful examination of the data obtained from the comparator arms of four trials shared via the Project Data Sphere portal revealed that two of the trials did not provide all the required survival data – a standard outcome measure in such trials and information that was crucial to our analysis. Further, we found different recording practices across trials. For example, while one trial captured patient death even if it occurred after withdrawal from the study, another only captured deaths occurring during the trial; these discrepancies can significantly affect the ability to conduct direct therapy comparisons.

In a second study, we set out to combine data from four clinical trials testing biologic therapies for the treatment of psoriasis, with the aim of identifying subgroups of patients with differing patterns of response. In this instance, two of the trials did not record symptomatic-level characterization of the disease, information that was captured by the other two trials. This impacted our ability to include such information in our models and our analysis plan had to be adjusted accordingly, possibly resulting in less than optimal description of the identified clinical response patterns.

Missing information, as well as differences in the information recorded across trials, may only be discovered after a significant amount of time is spent on data cleaning and manipulation; encumbering secondary data use and reducing research efficiency. The extent of such differences could vary, and as such so will the downstream effect on intended analyses.

## High complexity in trial design

**Fig. 1:**
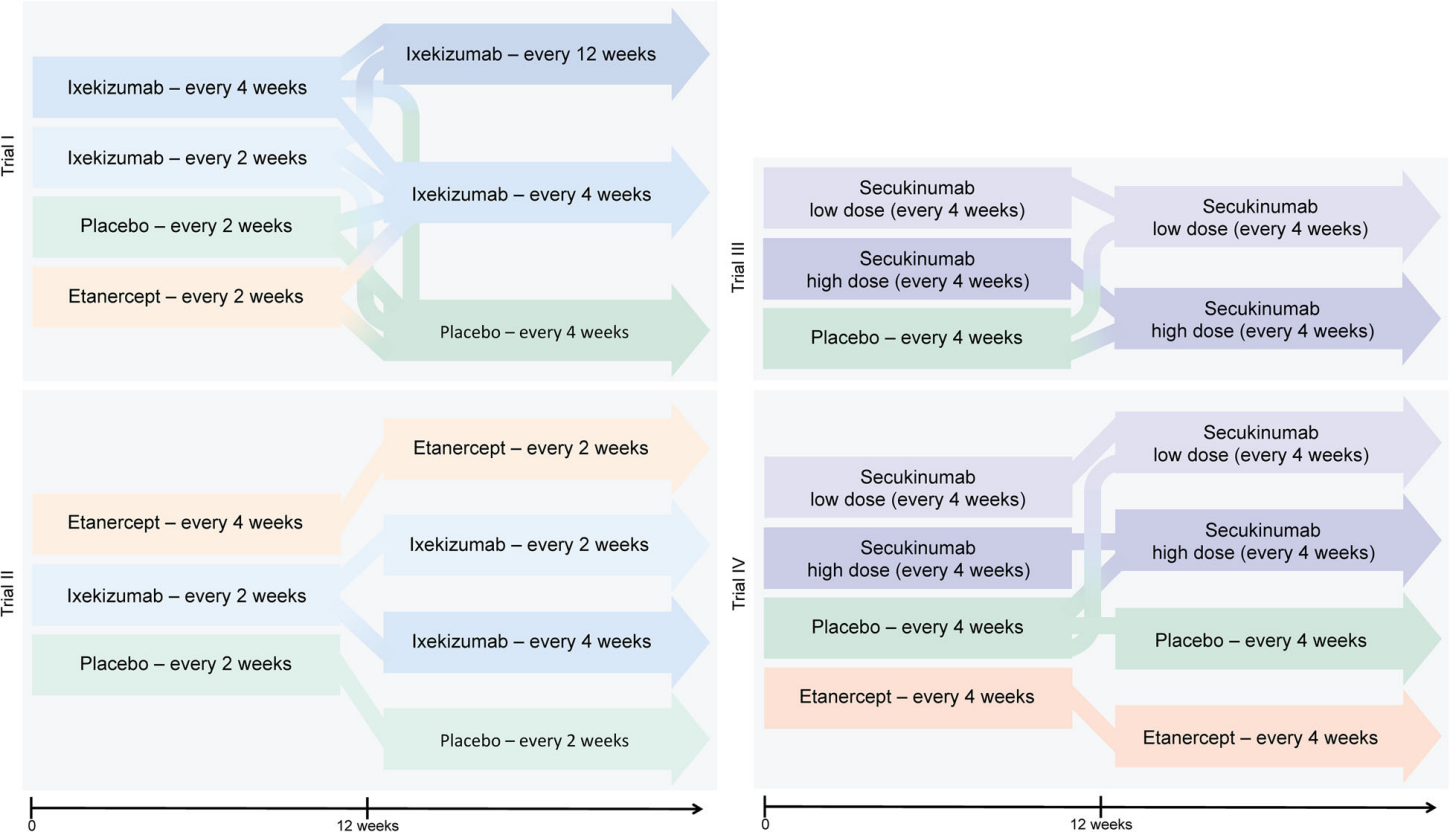
Different treatment protocols across four clinical trials of biologic therapies in psoriasis. All protocols were segmented into two parts, the first 12 weeks and then the rest of the duration of follow-up; with some arms continuing on the same treatment and others switching medications or dose

Our psoriasis patient-level meta-analysis additionally presented high trial-protocol complexity, potentially limiting our planned analyses or the methods by which they could be carried out. Across the four trials, a total of 19 different treatment protocols were used, with 10 of these originating from a single trial, designed with the purpose of testing the effect of changing treatment dose on patient outcome (Fig. 1). As a result, data across more than one trial could only be integrated for five treatment arms in which the entire set of protocols was identical, and patient baseline characteristics were similar enough to allow for pooling of the data. This substantially impacted the strength of our investigation.

## Overcoming complexities in trial design and data sharing

The level of complexity in the design, and ambiguity with regards to availability of information can compound intended secondary analyses to varying degrees. While accurate description of trials and the data being shared is an issue that in principle can be tackled, handling complexity in trial design is more challenging. The challenges that exist in the reuse of data are further exaggerated when data from multiple studies are reused and pooled; as with our examples. Re-analysis of data from a single trial may not always be impaired by limited understanding of the original study. In such cases, most aspects of the study design will remain similar for all participants and are thus less likely to influence the results. However, in a pooled analysis, which poses a special case of data reuse that is particularly useful for increasing sample sizes and for making comparisons that are otherwise not possible, even slight differences between pooled datasets could present a source of heterogeneity. Baseline differences between cohorts, differences in which patient characteristics and outcome measures are used and how they are recorded, as well as missing values, can all affect the planned analysis. A thorough understanding of all aspects of the studies, as well as selection of appropriate statistical methods, therefore, becomes imperative.

This added layer of complexity, introduced when data are combined across trials, requires that these pool-and-reuse studies themselves be carefully designed beforehand, very much like clinical trials. However, this careful design can only happen when highly detailed information about the original studies is provided. Projects, such as that described in Goldacre et al., look to link clinical trials with relevant trial documentation such as protocols, reports, and trial forms, as well as other literature of potential interest. This is a step in the right direction to provide researchers with the critical information needed when reusing trial datasets [7].

Nevertheless, even with existing barriers in the current state of trial data sharing, there is merit in reusing these often rich and much invested-in data. Our analysis of data, combined from eight prostate cancer clinical trials demonstrating the survival benefits of some standards of care over others [11], exemplifies potential gains from trial data pooling and reuse. In a second example, pooling data on placebo-treated patients across many failed trials in Alzheimer’s disease allowed for the identification of three trajectories of disease progression [12]; generating new hypotheses that would never have come to light in a standard trial.

It has been previously proposed that secondary users collaborate closely with primary clinical trialists to allow them to retain ownership and control of the uses of their data, but also to prevent secondary users from misunderstanding trial complexities and nuances [13]. This, however, is not always easy to achieve in practice. As suggested by Ohmann et al., involvement of data generators is not a necessity but primary data generators should have the option of being alerted about who requests access to their data and when [4]. A possible alternative is to get clinical specialists, experts in the specific field of medicine of focus, involved in the research, to advise and help inform analytical decisions. It may be worthwhile for data providers to require evidence of relevant clinician involvement in applications for access to trial data in a specific area of medicine.

A further consideration for clinical trial researchers may be that, if secondary analysis is considered an important outcome in itself, trial protocols should be designed with secondary analysis in mind. However the primary objectives of research cannot be forgone for the sake of secondary research potential. One possible solution would be to provide, prior to sharing of actual data, synthetic datasets that enable the design of pool-and-reuse studies by preserving the essential properties of the data.

A perhaps more sustainable solution is to impose standards for clinical trial data sharing. These have been proposed and lessons can be learned from efforts made to standardize electronic health records data, and recommendations made for non-commercial clinical trials. It may be the case that these need to be further developed and enforced, to provide detailed, complete data annotations, helping circumvent many data-related obstacles and allowing for more usable and utilitarian trial data sharing.

## Data Availability

The data that support the findings of this study are available from Project Data Sphere and from ClinicalStudyDataRequest.com but restrictions may apply to the availability of these data, which were used under license for the current study.

## Abbreviation

FHIR: Fast Health Interoperability Resources
